# 

**Published:** 1905-09

**Authors:** 


### BOOKS RECEIVED.

Physical Diagnosis. By Richard C. Cabot, M. D., Instructor in Medicine in Harvard University. Third edition, revised and enlarged. Small octavo, pp. xxii.-577. Illustrated. New York: William Wood & Co. 1905.

Ophthalmic Neuromyology. A Study of the Normal and Abnormal Actions of the Ocular Muscles from the Brain Side of the Question. By G. C. Savage, M.D., Professor of Ophthalmology in the Medical Department of Vanderbilt University. Small 8vo, pp. 221. Illustrated. Nashville: Keelin-Williams Printing Co. 1905.

A Practical Treatise on Sexual Disorders in the Male and Female. By Robert W. Taylor, A.M., M.D., Clinical Professor of Genitourinary and Venereal Diseases in the College of Physicians and Surgeons (Columbia University), New York. Third edition, enlarged and revised. Octavo, 575 pages with 130 engravings and 16 colored plates. Philadelphia and New York: Lea Brothers & Co. 1905. Cloth, $3.00, net.

A Text-Book of Physiology, Normal and Pathological. For Students and Practitioners of Medicine. By Winfield S. Hall, Ph.D., M.D., (Leipzig), Professor of Physiology, Northwestern University Medical School, Chicago. Second edition, revised and enlarged. Octavo, 795 pages, with 339 engravings and three full-page colored plates. Philadelphia and New York: Lea Brothers & Co. 1905. Cloth, $4.00, net.

Color-Vision and Color-Blindness. A Practical Manual for Railroad Surgeons. By J. Ellis Jennings, M.D. (University of Pennsylvania). Formerly Clinical Assistant Royal London Ophthalmic Hospital; Professor of Diseases of the Eye, Medical Department Barnes University, St. Louis. Second edition. Revised with illustrations. Pp. 132. Small octavo. Philadelphia: F. A. Davis Company. (Price, $1.00, net).

International Clinics. A Quarterly of Illustrated Clinical Lectures and especially prepared articles on Treatment, Medicine, Surgery, Neurology, Pediatrics, Obstetrics, Gynecology, Orthopedics, Pathology, Dermatology, Ophthalmology, Otology, Rhinology, Laryngology, Hygiene and other topics of interest to students and practitioners. By leading members of the medical profession throughout the world. Edited by A. O. J. Kelly, A.M., M.D., Philadelphia. Volume II. Fifteenth series. 1905. Philadelphia and London: J. B. Lippincott Co. (Cloth, $2.00).

A Syllabus of Materia Medica. By Warren Coleman, M.D., Professor of Clinical Medicine in Cornell University Medical College. Second edition. New York: William Wood & Co. 1905.

Moustiques et Fievre jaune, par A. Chantemesse, professeur d'hygiène á la Faculté de médecine de Paris et F. Borel, directeur de la 2e circonscription sanitaire maritime, 1 vol. in-18 de 96 pages, avec figures, cart.: 1 fr. 50. Paris: J. B. Bailliere et Fils. 1905.

Green's Pathology. Tenth edition. A Textbook of Pathology and Pathological Anatomy. By T. Henry Green, M.D., F.R.C.P., Consulting Physician to Charing Cross Hospital, London. New (tenth) edition. Thoroughly revised by W. Cecil Bosanquet, A.M., M.D., F.R.C.P., Assistant Physician to Charing Cross Hospital. Octavo, 606 pages, 348 engravings and a colored plate. Philadelphia and New York: Lea Brothers & Co., Publishers. 1905. (Cloth, $2.75 net.)

Jackson on the Skin. A Ready Reference Handbook on Diseases of the Skin. By George Thomas Jackson, M.D., Chief of Clinic and Instructor in Dermatology, College of Physicians and Surgeons (Columbia University), New York. Fifth edition, enlarged and thoroughly revised. In one 12mo volume of 676 pages, with 91 engravings and 3 colored plates. Philadelphia and New York: Lea Brothers & Co., Publishers. 1905. (Cloth, $2.75 net.)

Manual of the Diseases of the Eye. For Students and Practitioners. By Charles H. May, M.D., Chief of Clinic and Instructor in Ophthalmology, College of Physicians and Surgeons, Medical Department of Columbia University, New York, 1890-1903; Ophthalmic Surgeon to the City Hospital, Randall's Island, New York, etc., etc. Fourth edition, revised, with 360 illustrations, including 21 plates and 60 colored figures. Small octavo, pp. viii.-391. New York: William Wood & Co. 1905. (Price, $2.00 net.)

